# Effectiveness of Probiotics and Prebiotics Against Acute Liver Injury: A Meta-Analysis

**DOI:** 10.3389/fmed.2021.739337

**Published:** 2021-09-21

**Authors:** Sheng Xu, Min Zhao, Qinjian Wang, Zhihua Xu, Binhui Pan, Yilang Xue, Zebin Dai, Sisi Wang, Zhanxiong Xue, Fangyan Wang, Changlong Xu

**Affiliations:** ^1^Department of Gastroenterology, The Second Affiliated Hospital and Yuying Children's Hospital of Wenzhou Medical University, Wenzhou, China; ^2^Department of Pathophysiology, School of Basic Medicine Science, Wenzhou Medical University, Wenzhou, China

**Keywords:** acute liver injury, probiotics, prebiotics, gut microbiota, gut-liver axis, meta-analysis

## Abstract

**Background and Aims:** Acute liver injury (ALI) is a clinical syndrome characterized by rapid loss of liver function, which may progress to life-threatening liver failure. We conducted this meta-analysis to examine the evidence on the effects of probiotics or prebiotics on ALI.

**Methods and Results:** Several databases, including PubMed, EMBASE, and Cochrane Library, were scrutinized from the inception through February 2021 by combining key search terms, yielding 26 eligible studies, which concluded that modulation of gut microbiota significantly decreased aspartate transaminase [standardized mean difference (SMD): −1.51, 95% confidence interval (CI): −2.03 to −1.00], alanine aminotransferase (SMD: −1.42, 95% CI: −1.85 to −0.98), and bilirubin (SMD: −0.91, 95% CI: −1.33 to −0.49). In addition, administration of probiotics or prebiotics also promoted proliferation of *Bifidobacterium* (SMD: 1.21, 95% CI: −0.18 to 2.60) and inhibited *Enterococcus* (SMD: −1.00, 95% CI: −1.39 to −0.61), contributing to lower levels of endotoxin (SMD: −2.14, 95% CI: −2.91 to −1.37). Tight junction protein ZO-1 (SMD: 1.95, 95% CI: 0.14 to 3.76) was upregulated after intervention, thereby reducing bacterial translocation to the liver [odds ratio (OR) = 0.23, 95% CI: 0.13–0.44] and mesenteric lymph node (OR = 0.14, 95% CI: 0.08 to 0.26), with decreased tumor necrosis factor-α (SMD: −2.84, 95% CI: −3.76 to −1.93) and interleukin-6 (SMD: −2.62, 95% CI: −4.14 to −1.10). Oxidative stress was also relieved by reducing malondialdehyde (SMD: −1.83, 95% CI: −2.55 to −1.10) while elevating superoxide dismutase (SMD: 1.78, 95% CI: 1.00–2.55) and glutathione (SMD: 1.83, 95% CI: 0.76–2.91).

**Conclusion:** Our findings suggest that probiotics and prebiotics could be a promising therapeutic strategy in ALI and possess a potential for clinical applications.

**Systematic Review Registration:**https://www.crd.york.ac.uk/PROSPERO/display_record.php?RecordID=255888, CRD42021255888.

## Introduction

Acute liver injury (ALI) is defined as a rapid degeneration of liver biochemistry within 6 months in patients with no prior liver disease (1). With the further loss of liver function and impaired metabolism of toxic substances, patients with ALI may develop jaundice, coagulation dysfunction, and hepatic encephalopathy, marking the progression to acute liver failure characterized by multisystem complications and high mortality of up to 80% (2). Liver transplantation is considered to be the only definitive treatment in the stage of acute liver failure (3), but it is limited by a shortage of graft availability, which creates the urgent need to seek complementary and promising therapies to prevent the progression of ALI.

For decades, emerging evidence has indicated the tight connections between the gut microbiota and liver injury, continuously enriching the theory of the gut–liver axis. In the context of disease, disturbances of the intestinal barrier increase the portal influx of bacteria or their metabolites to the liver, causing dysfunction in the metabolism of bile acids, and promote systemic inflammation and liver damage, which in turn intensifies gut dysbiosis (4). In addition, pathogen-associated molecular patterns, such as lipopolysaccharides (LPS), from certain intestinal bacteria stimulate nuclear factor kappa B (NF-κB) *via* toll-like receptors (TLRs) and nod-like receptors, resulting in the production of inflammatory mediators and chemokines, with the activation of stellate cells involved in fibrosis progression (5).

Microbial agents are typically referred to as probiotics and prebiotics, which are defined as living microorganisms that contribute health-promoting benefits to the host and nondigestible food ingredients that are selectively utilized by host microorganisms to stimulate health benefits, respectively. Studies have shown that both strategies of regulating intestinal microbiomes could exert a curative effect through the gut–liver axis. Since the regulation of intestinal microecology has long been known to possess definite therapeutic efficacy in the treatment of chronic liver diseases, including arresting the progression of hepatic fibrosis and preventing the occurrence of hepatic encephalopathy (4), the relationship between intestinal flora and ALI has received increasing attention. It has been previously observed that correction of the gut microbiota composition significantly attenuated t-BHP-induced liver injury by intensifying gut barrier with increased expression of tight junction proteins, including claudin-1, occludin, and zonula occludens (ZO-1), thus suppressing bacterial translocation (BT) and the expression of LPS-stimulated proinflammatory mediators. In addition, beneficial bacteria were verified to regulate tumor necrosis factor-α (TNF-α) expression by inhibiting the TLR4-associated NF-κB signaling pathway and correcting the Th17/Treg imbalance *via* the mediation of innate immune cells.

However, the effectiveness of probiotics and prebiotics on ALI has not been systematically evaluated. Therefore, the purpose of this study was to comprehensively analyze and quantify credible evidence from published studies, seeking to offer valuable information for future research on the treatment of ALI.

## Methods

### Protocol Registration

This meta-analysis was registered on PROSPERO (ID: CRD42021255888; https://www.crd.york.ac.uk/PROSPERO/display_record.php?RecordID=255888).

### Search Strategy

We performed this meta-analysis in accordance with the recommended Preferred Reporting Items for Systematic Reviews and Meta-analyses guidelines (6). A comprehensive search of English literature published up to January 2021 was conducted in PubMed, EMBASE, the Cochrane Central Register of Controlled Trials, and clinicaltrials.gov databases using combinations of the Mesh terms and synonyms, including “probiotics,” “yogurt,” “yogurt,” “*Lactobacillus*,” “*Bifidobacterium*,” “*Enterococcus*,” “*Streptococcus*,” “*Saccharomyces*,” “*Lactococcus*,” “*Bacillus*,” “prebiotics,” “inulin,” “oligosaccharide,” “galactose oligosaccharide,” and “fructose oligosaccharide” for microbial agents, and “liver injury,” “toxic hepatitis,” “drug-induced liver injury,” and “chemical-induced liver injury” for ALI. Additional studies were also checked by a hand search of all the references of the retrieved articles. After eliminating duplicates, we screened the titles, abstracts, and full texts sequentially for eligible records according to the selection criteria. Any disagreement was solved through discussion.

### Study Selection

The participants, intervention, comparison, outcome, and study design (PICOS) principle was followed during the literature screening. Studies that met the following inclusion criteria were considered for selection: Participants (P): patients or animal models of ALI regardless of the cause. It should be noted that no clinical studies were available at the end of the selection process, and all the literature involved was on animal research; therefore, the objectives were finally limited to animal models; Intervention (I): the intervention group received microbial agents, such as prebiotics or probiotics regardless of dosage, route, and duration; Comparison (C): control group without receiving microbial agents; Outcome (O): the studies should at least measure one of the following indicators: alanine transaminase (ALT), aspartate aminotransferase (AST), bilirubin, TNF-α, interleukin-6 (IL-6), IL-10, cholesterol, triglyceride, BT, endotoxin, malondialdehyde (MDA), superoxide dismutase (SOD), glutathione (GSH), or ZO-1; Study design (S): randomized controlled studies. Studies with the occurrence of acute liver failure during the trial or unavailable data were excluded.

### Quality Evaluation and Data Extraction

Since all clinical studies were removed during the screening process, with only preclinical studies included according to the selection criteria, the methodological quality was evaluated based on the rules detailed in systematic review centre for laboratory animal experimentation's (SYRCLE) risk of bias tool (7). The following information was extracted from the literature involved: first author, publication year, animal species, sample size, modeling methods, intervention and dosage, route and duration, and comparison and outcome indicators. Disagreements were addressed through consensus. Getdata 2.20 was used in the data extraction.

### Statistical Analyses

Pooled mean differences and standard deviations with 95% confidence intervals (CIs) were applied to determine differences in outcomes of continuous variables, with results of the dichotomous variables reported as odds ratio (OR) and 95% CI. Heterogeneity was assessed by using the Cochran *Q*-test and *I*^2^ statistics (I^2^ < 25%, low heterogeneity; 25–50%, moderate heterogeneity; *I*^2^ > 50%, high heterogeneity). A random-effects model will be performed due to the exploratory nature of the animal studies. A value of *p* < 0.05 was considered statistically significant. Subgroup analysis was also performed for indicators with more than 10 included studies according to the type of microbial agents, bacterial strain, animal model, and modeling method to explore the source of heterogeneity. We also used the meta-regression to detect where the potential factor for heterogeneity originated. Sensitivity analysis was conducted to identify the studies that significantly affected the findings by excluding studies from the analysis one by one to gauge the robustness of our results. Publication bias was estimated quantitatively using Egger's test. A contour-enhanced funnel plot with the “trim and fill” method was obtained as an aid to differentiate asymmetry caused by publication bias or other factors such as heterogeneity (34). If the missing studies were in the nonsignificant area, the asymmetry was due to publication bias. Otherwise, the observed asymmetry could be attributed to factors other than publication bias. Statistical analyses were performed using R (version 4.0.0) and the R Studio software (R Studio, Boston, MA, USA).

## Results

### Identification of Relevant Study

**Figure 1:**
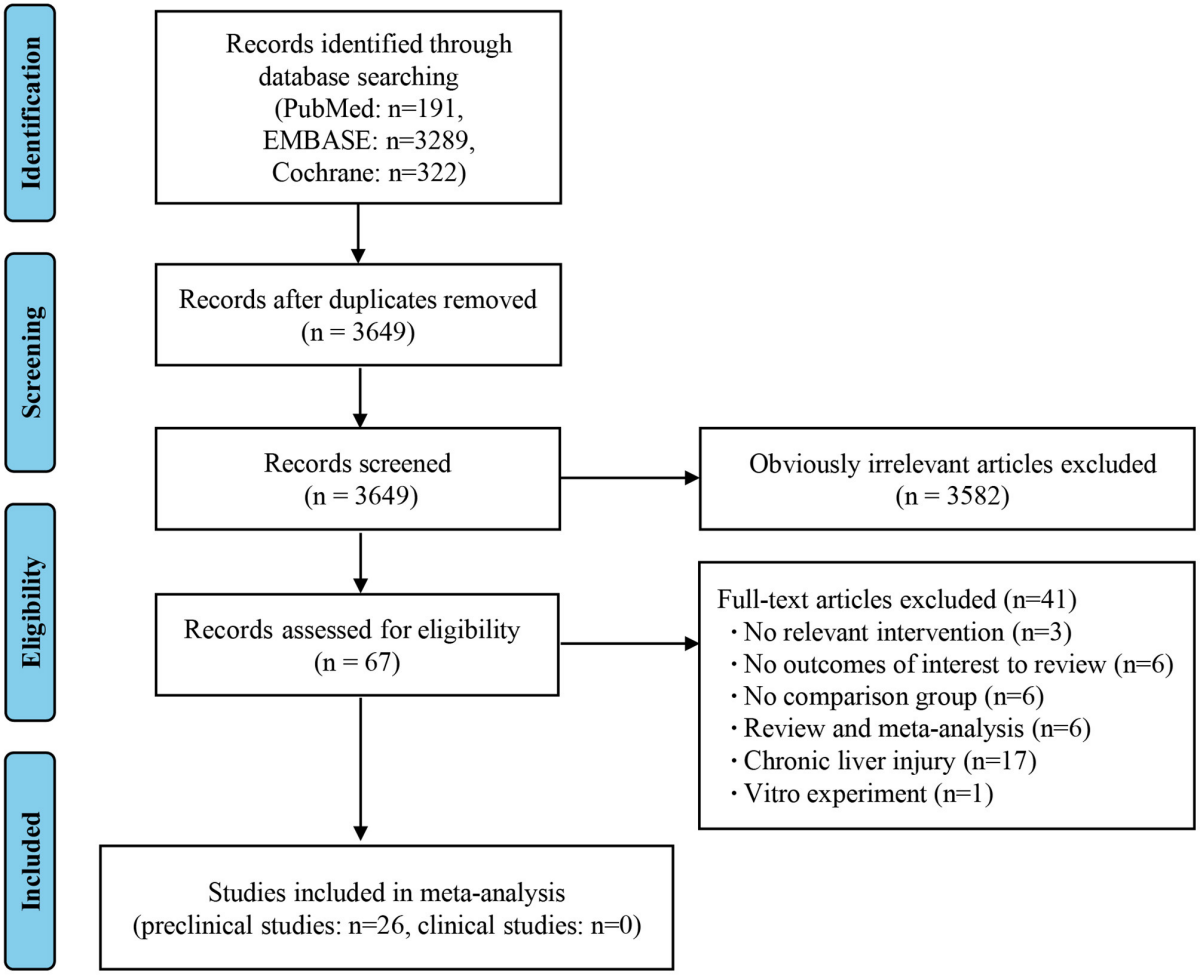
Flow diagram based on the Preferred Reporting Items for Systematic Reviews and Meta-Analyses (PRISMA).

As shown in the PRISMA flow diagram, a total of 3,802 studies were initially retrieved by searching the databases, which reduced to 3,649 after the deletion of duplicates. Preliminary screening of the titles and abstracts reduced the number to 67 articles after the exclusion of 3,582 studies. A further 41 articles were rejected based on a detailed full-text evaluation, with 26 studies ultimately being eligible for the data extraction and analysis. All the literature involved was on animal studies, as there were no clinical studies that met the selection criteria (Figure 1) (8–33).

### Study Characteristics and Quality Assessment

Table 1 shows the characteristics of the 26 selected studies. The rodent animal model was applied as the research objects in all the literature involved, most of which used Sprague–Dawley rats. In addition, the modeling methods of ALI were different, among which the intraperitoneal injection of D-galactosamine had the highest frequency of utilization, followed by CCI4 and LPS. Probiotics, such as *Lactobacillus* and *Bifidobacterium*, were more commonly used in the intervention group compared to prebiotics (including lactulose and inulin), which were employed by only six studies. Oral gavage was the most commonly used route of drug delivery, while transrectal administration through a rectal tube was used by only two studies. Saline was used as the control in most of the studies, while a standard diet was used by Nardone, G.

**Table 1:** Characteristics of included studies investigating the effects of probiotics or prebiotics on ALI.

**References**	**Animal**	**Sample size**	**Modeling methods**	**Intervention and dosage**	**Route and duration**	**Comparison**	**Outcome indicators**
Adawi et al. (8)	Male Sprague–Dawley rats, weighing 200–300 g	30	Intraperitoneal injection of D-Ga1N on the 8th day	*Lactobacillus acidophilus* NM1, *Lactobacillus rhamnosus* ATCC 53103, *Bifidobacterium animalis* NM2, *Lactobacillus rhamnosus* DSM 6594, *Lactobacillus plantarum* DSM 9843 (3 × 10^9^ CFU)	Rectally, for 8 days	Normal saline	ALT↓, AST↓, Bilirubin↓, and BT↓
Adawi et al. (9)	Male Sprague–Dawley, weighing 200–300 g	54	Intraperitoneal injection of D-Ga1N (1.1 g/kg body weight) on the 8th day	Probiotics: *Lactobacillus plantarum* DSM 9843 (strain 299v) (3 × 10^9^ CFU)	Rectally, for 8 days	Normal saline	ALT↓, AST↓, BT↓, Cecal Bacterial Count↑, Colonic Bacterial Count↑, Intestinal Mucosal Nucleotides, RNA, and DNA Content↓
Han et al. (10)	Male Imprinting Control Region (ICR) mice, 20–22 g	90	Orally treated with 0.025 mL CCI4/kg or intraperitoneally treated with 1.5 mmol t-BHP/kg	Lactic acid bacteria at dose of 0.5 g/kg and 2 g/kg (L7, 0.5 g/kg *L. brevis* HY7401; H7, 2 g/kg *L. brevis* HY7401; LC, 0.5 g/kg *L. acidophilus* CSG; HC, 2 g/kg *L. acidophilus* CSG; L8, 0.5 g/kg *B. Iongum* HY8001; H8, 2 g/kg *B. Iongum* HY8001)	Orally, for 3 days	Saline	ALT↓, AST↓, and ß-glucuronidase activities↓
Jia and Zhang (11)	Male Sprague-Dawley rats weighing 220–250 g	36	Intraperitoneal injection of TAA (200 mg/kg in normal saline, purity >99%) every 24 h for two consecutive days	Probiotics: Golden Bifid (highly concentrated combination of probiotic) dissolved in 2 ml of normal saline; Prebiotics: Lactulose (8 ml/kg)	Orally, once a day for 8 d (from the 5th day before the experiment to the 3rd day of the experiment)	Tap water *ad libitum*	Endotoxin↓, ALT↓, AST↓, Albumin↓, and TB↓
Kasravi et al. (12)	Sprague–Dawley rats	48	Intraperitoneal injection of 1.1 g/kg body weight of D-Ga1N on the 7th day	Probiotics: 5 ml suspension *Lactobacillus reuteri* R2LC; *Lactobacillus plantarum* DSM 9843 (0.5-−1.0 × 10^9^/ml)Prebiotics: Lactulose (5 ml/day of 20% lactulose solution)	Orally, for 7 days	Physiologic saline	ALT↓, AST↓, Intestinal microflora↑, BT↓, and Mucosal DNA/RNA↑
Li et al. (13)	Sprague–Dawley rats	48	Intraperitoneal injection of 1.1 g/kg body weight of D-GalN on day 7	2 ml/d (2.0 × 10^10^ CFU/ml resuspended in physiologic saline) of living *Bifidobacterium Catenulatum* ZYB0401 and *Lactobacillus Fermentum* ZYL0401	Gavage, for 7 days	Physiologic saline	ALT↓, AST↓, Endotoxin↓, BT↓, and Terminal Ileum microflora↑
Nardone et al. (14)	Male Wistar rats weighing 200–250 g	54	Ischemia-reperfusion (I/R) (30 min ischemia and 60 min reperfusion)	3 × 10^7^ CFU live *Lactobacillus paracasei* F19 (LP-F19)	Orally, for 8 weeks	Standard diet	ALT↓, AST↓, LPS↓, TNF-α↓, IL-1β↓, IL-6↓, MDA↓, and Ileal mucosa bacteria counts↓
Nicaise et al. (15)	Ten–twelve-week-old male C57BL/6 mice and 4-week-old male Lewis rats	16	Injected intraperitoneally TAA at 250 mg/kg body weight in NaCl 0.9% on day 0 and 1	Probiotics: 100 μl of 10^7^, 10^8^, or 10^9^ CFU of viable *Lactobacillus plantarum* strains; Prebiotics: Lactulose (6 g/kg)	Orally and intrarectally, for 3 days	Normal saline	ALT↓, AST↓, Blood Ammonia↓, Liver injury↓, and Survival rate↑
Osman et al. (16)	Sprague–Dawley rats weighing 200 g	36	Intraperitoneal injection of D-Ga1N 1.1 g/kg bodywt and 10 μl endotoxin on the 8th day	3 ml (10^8^ CFU/ml) of the *Lactobacillus* or the *Bifidobacterium* tested bacterial strain	Oral administration (twice daily for 8 days)	Normal saline	ALT↓, Bilirubin↓, TNF-α↓, and Myeloperoxidase (MPO)↓
Park et al. (17)	Six-week-old male ICR mice	54	An acute ethanol dose of 3 g/kg of body weight diluted in water	*Lactobacilli fermentum* strain OCS19 (10^9^ CFU/kg)	Orally by gavage once daily for 7 days	Water	ALT↓, AST↓, and Triglyceride↓
Rishi et al. (18)	Wistar rats, 6–8 weeks old weighing 150–200 g	56	LPS (10 mg/kg body weight, prestandardized dose) intraperitoneally on day 11	Probiotics: 1 ml of *Lactobacillus plantarum* (containing 10^10^ CFU)	Oral gavage, daily for 10 days	Normal saline	ALT↓, AST↓, Kupffer cell death↓, Hepatic nitrite levels↓, and TNF-α↓
Rishi et al. (19)	BALB/c mice (18–22 g)	64	0.2 mL of S. typhimurium (2.5 × 10^7^ CFU/mL) on day 3	Probiotics: 0.2 mL of *Lactobacillus acidophilus* (1 × 10^10^ cells per mouse); Prebiotics: Inulin (0.2 ml of 10 ml/mg)	Orally for 10 days	Normal saline	ALT↓, AST↓, BT↓, Malondialdehyde↓, GSH and SOD↓, and Nitrite↓
Sharma et al. (20)	Male Wistar rats weighing 200 ± 10 g	42	Acetaminophen (APAP) (1 g/kg of body weight in 0.5% CMC) for 14 days	*Enterococcus lactis* IITRHR1 (2 × 10^10^ CFU per gram of IITRHR1 lyophilized powder; 10^7^, 10^8^, and 10^9^ CFU)	Gavage for 7 days	The vehicle (0.5% CMC)	ALT↓, AST↓, Bilirubin↓, SOD activity↑, CAT activity↑, GPx activity↑, GST activity↑, Redox ratio↑, Lipid peroxidation and protein oxidation↓, Bax↓, and Bcl2↑
Wang et al. (21)	Male Wistar rats weighting 120–140 g of 5–6 weeks old	48	Injected intraperitoneally with 50 μg/kg LPS and 300 mg/kg D-GalN on the 31 days	*Lactobacillus casei* Zhang 1 ml (1 × 10^9^ CFU/ml)	Gavaged for 30 days	Saline	Survival rates↑, ALT↓, AST↓, MDA↓, SOD activity↑, NO/iNOS expression↓, TNF-α↓, and TLR4↑
Xing et al. (22)	Specific pathogen-free male Sprague–Dawley rats weighting 190–210 g	54	Hepatic ischemia-reperfusion (I/R) injury	4 ml/day of living *Bifidobacterium Catenulatum* ZYB0401 containing 1.2 × 10^9^ CFU 4 ml/day of living *Lactobacillus Fermentum* ZYL0401 containing 1.2 × 10^9^ CFU, 4 mL/day mixed suspension of *B. Catenulatum* ZYB0401 (1.2 × 10^9^ CFU) and *L. Fermentum* ZYL0401 (1.2 × 10^9^ CFU)	Gavaged for 7 days	Physiologic saline	ALT↓, AST↓, Plasma endotoxin↓, TNF-α↓, MDA↓, SOD activity↑, BT↓, and Mucosal integrity↑
Shi et al. (23)	Male pathogen-free Sprague–Dawley rats weighing 250–350 g	98	Subcutaneous injection of a 50% (V/V) CCl4 solution in olive oil twice per week at a dose of 2 ml/kg	*Lactobacillus salivarius* LI01; *Pediococcus pentosaceus* LI05; *Clostridium butyricum* MIYAIRI; *Bacillus licheniformis* Zhengchangsheng; *Lactobacillus rhamnosus* GG (3 × 10^9^ CFU/ml)	Gavage, once daily for 13 weeks	Normal saline	ALT↓, AST↓, Bilirubin↓, BT↓, IL-6↑, TNF-α↓, IL-10↓, Survival time↑, and ZO-1↑
Moratalla et al. (24)	Female Balb/c mice weighing 18–20 g	52	Weight-controlled doses of CCl4 intragastrically administered for 12 weeks	*Bifidobacterium pseudocatenulatum* CECT7765 (× 10^9^ CFU daily)	Intragastrically, for a week	Placebo (vehicle)	BT↓, TNF-α↓, IL-10↑, MDA↓, and Hydroxyproline↓
Peng and Jiang (25)	Male Kunming mice (weighing 18–22 g)	30	At 16 days, intraperitoneally injected with LPS at 4 mg/kg	*Lactobacillus plantarum* NDC 75017 (1 × 10^9^ CFU/ml)	Oral gavages for 15 days	Normal saline	ALT↓, AST↓, MOD↓, NO↓, SOD↑, GSH↑, iNOS activity↓, TNF-α↓, IL-10↑, and IL-6↓
Jin et al. (26)	C57BL/6 female and male mice (6–8 weeks old)	28	Intraperitoneal injection of LPS	*Lactobacillus fermentum* ZYL0401 (2 × 10^10^ CFU)	Gavages, for 10 consecutive days	Phosphate-buffered saline (PBS)	ALT↓, TNF-α↓, IL-10↑, Ileal COX2 mRNA↑, and MPO↑
Liu et al. (27)	Male ICR mice (25–30 g)	30	Intraperitoneally with a lethal dose of CCl4 (3 ml/kg).	*Clostridium butyricum* (5 × 10^8^ CFU)	Gavages, for 5 days	Normal saline	Survival rate↑, ALT↓, AST↓MDA↓, SOD↑, CAT↑, NRF2↑, IL-6↓, and IL-1β↓
Chen et al. (28)	Kunming mice (male, 6 weeks old)	60	CCl4 was intraperitoneally injected into the mice at 14th day	*Lactobacillus plantarum*; *Lactobacillus fermentum* (10^9^ CFU/kg)	Gavages, for 14 days	Normal saline	ALT↓, AST↓, MDA↓, SOD↑, GSH↑, Survival rate↑, TNF-α↓, and IL-1β↓
Jang et al. (29)	Male C57BL/6 (21–23 g, 6 weeks old)	42	Intraperitoneally treated with 1.5 mmol t-BHP/kg	*Lactobacillus plantarum* LC27 (1 × 10^9^ CFU/mouse); *Bifidobacterium longum* LC67 (2 × 10^9^ CFU/mouse)	Orally, once a day for 3 days	Vehicle	TNF-α↓, IL-1β↓, IL-10↑, ALT↓, AST↓, and BT↓
Cui et al. (30)	Male C57BL/6 mice (20 ± 2 g body weight, aged 8 weeks)	24	Intraperitoneally injected with 10 mg/kg LPS	*Lactobacillus reuteri* ZJ617; *Lactobacillus rhamnosus* GG (10^9^ CFU)	Orally, daily for 2 weeks	Sterile PBS	ALT↓, AST↓, TNF-α↓, IL-10↑, L-6↑, ZO-1↑, Occludin↑, Claudin3↑, and Bax↓
Ding et al. (31)	Male 2–22-week-old Sprague–Dawley rats	60	CLP-Induced Sepsis	*Lactobacillus rhamnosus* GG (1 × 10^9^ CFU/ml)	Oral gavages	Sterile water	ALT↓, AST↓, TNF-α↓, Endotoxin↓, Liver injury severity↓, and IL-6↓
Li et al. (32)	Male germ-free Sprague–Dawley rats weighing 250–320 g	20	Intraperitoneal injection of D-Ga1N on the 15th day	*Bifidobacterium adolescentis* CGMCC 15058 (3 × 10^9^ CFU/ml)	Gavages, for 14 days	Normal saline	ALT↓, TNF-α↓, IL-10↑, IL-6↓, and BT↓
Wang et al. (33)	Male Sprague–Dawley rats (250–350 g)	18	D-GalN was injected intraperitoneally on the 8th day	*Bifidobacterium longum* R0175 (3 × 10^9^ CFU/ml)	Orally administered, for 7 days	Sterile normal saline	ALT↓, AST↓TNF-α↓, IL-10↓, IL-6↓, BT↓, GGT↓, and DPGA↓

*↑ and ↓ represent increased or decreased outcome indicators in the treatment group compared with control group, respectively. CFU, Colony-forming unit; CMC, Carboxy methyl cellulose; D-Ga1N, D-galactosamine; GDPA, Glycylproline dipeptidyl aminopeptidase; GGT, Gamma-glutamyltransferase; GPx, Glutathione peroxidase; ICR, Imprinting Control Region; iNOS, Inducible nitric oxide synthase; MPO, Myeloperoxidase; NO, Nitric oxide; TAA, Thioactamide*.

Two reviewers independently evaluated the methodological quality by employing SYRCLE's risk of bias tool. Each item was judged as “low risk,” “unclear,” or “high risk.” Blinding was the major contributor to bias; the blinding and allocation bias were unclear in most studies, as specific details of relevant information were often not provided. All studies were scored as having a low risk of bias in reporting bias. Overall, the assessment result was similar across the studies used in this meta-analysis with high quality and low risk of bias (Supplementary Table 1). Discrepancies during the process of quality assessment were solved through discussion.

### Effect of Microbial Agents on Liver Biochemical Indicators

**Figure 2:**
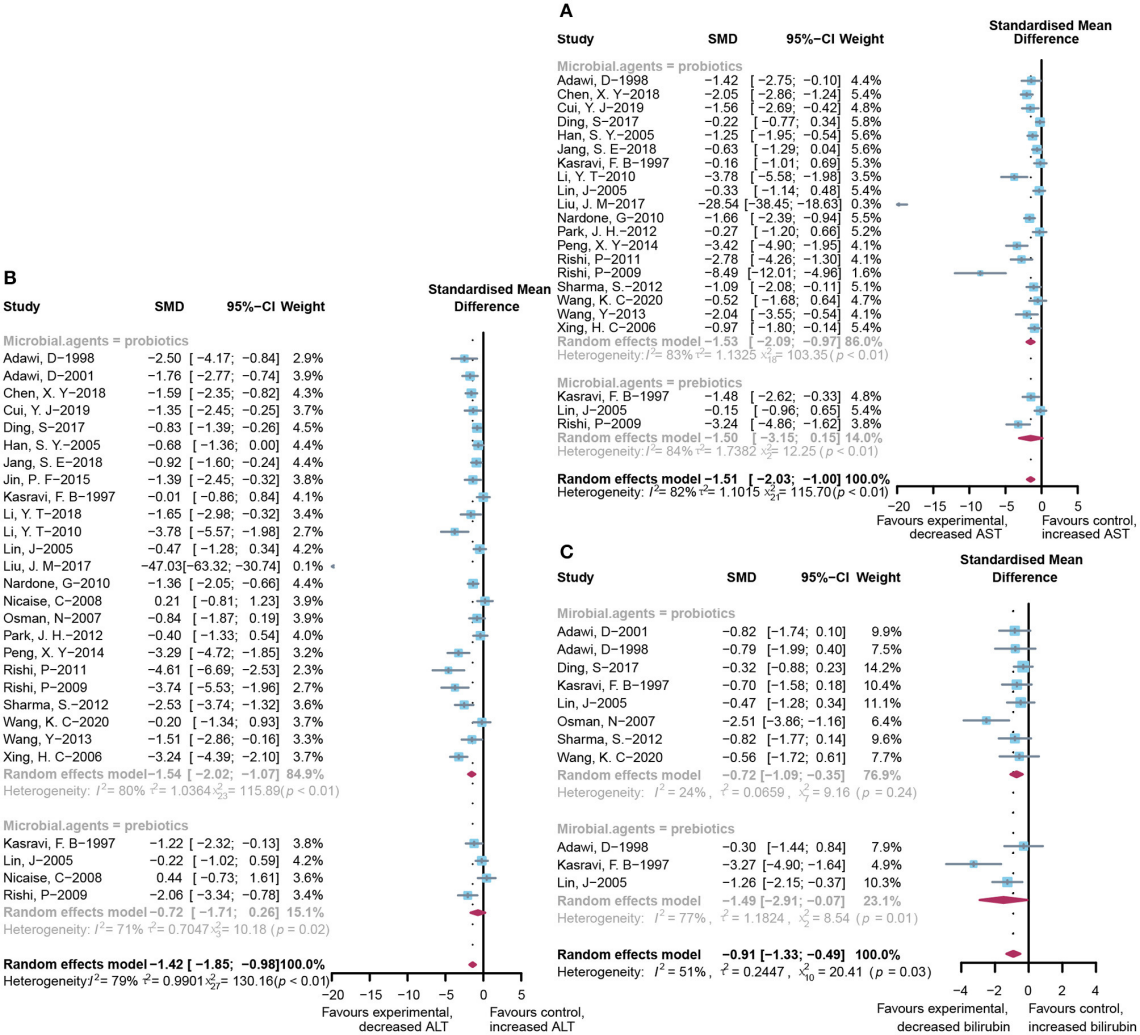
Effectiveness of microbial agents on liver biochemical index. **(A)** The effect of microbial agents on AST, **(B)** ALT, and **(C)** bilirubin.

**Figure 3:**
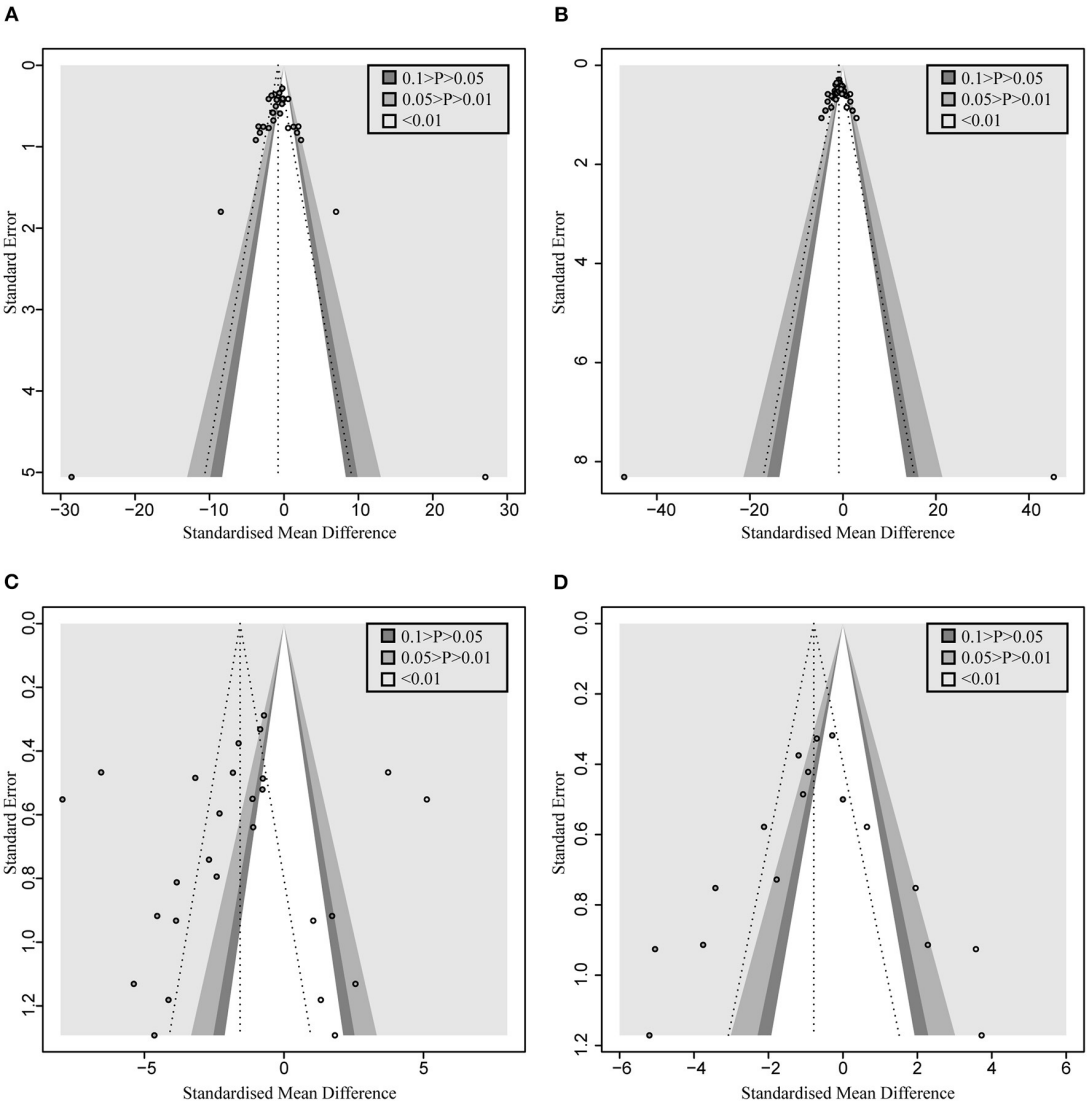
Contour-enhanced funnel plot with trim-and-fill method. **(A)** AST. **(B)** ALT. **(C)** TNF-α **(D)** MDA. If the missing studies were in the nonsignificant area, the asymmetry was due to publication bias. Otherwise, the observed asymmetry could be attributed to factors other than publication bias.

A total of 22 and 27 preclinical studies evaluated the effect of microbial agents on the liver function indicators, AST and ALT, respectively. Our pooled analysis showed a significant difference between the microbial intervention group and those treated with placebo in AST (standardized mean difference (SMD): −1.51, 95% CI: −2.03 to −1.00, *I*^2^ = 82%, and *p* < 0.01) and ALT (SMD: −1.42, 95% CI: −1.85 to −0.98, *I*^2^ = 79%, and *p* < 0.01; Figures 2A,B), indicating the positive effects of microbial regulation on normalizing AST and ALT in an ALI model. Owing to the significant heterogeneity, subgroup analyses were carried out to find out potential sources. Heterogeneity was slightly altered after dividing into probiotics (AST—SMD: −1.53, 95% CI: −2.09 to −0.97, *I*^2^ = 83%, and *p* < 0.01; ALT—SMD: −1.54, 95% CI: −2.02 to −1.07, *I*^2^ = 80%, and *p* < 0.01) and prebiotics (AST—SMD: −1.50, 95% CI: −3.15 to 0.15, *I*^2^ = 84%, and *p* < 0.01; ALT—SMD: −0.72, 95% CI: −1.71 to 0.26, *I*^2^ = 71%, and *p* = 0.02), but reduced in the subgroup animal model of AST (Table 2), indicating that it might be the source of heterogeneity, which was attested using meta-regression (Table 3). Subgroup analysis and meta-regression of ALT failed to find the source of heterogeneity (Supplementary Tables 2A, 3A). Publication bias existed in Egger's test (both *p* < 0.01). Although there is an obvious asymmetry in the contour-enhanced funnel plot (Figures 3A,B), it was proven that the asymmetry was caused by factors other than publication bias after using the trim-and-fill method. Sensitivity analysis confirmed the robustness of the study (Supplementary Figures 1A,B). Results from the pooled random-effects model of eight selected studies with the relevant data showed a decrease in bilirubin level after being treated with microbial agents (SMD: −0.91, 95% CI: −1.33 to −0.49) with moderate heterogeneity (*I*^2^ = 51%, *p* = 0.03; Figure 2C), which further demonstrated the protective effect of microbial agents on the liver.

**Table 2:** Subgroup analysis of AST.

	**No. of studies**	**SMD (95% CI)**	***I*^**2**^ (%)**	***p*-value**
**Microbial agents**				
Probiotics	19	−1.53 (−2.09, −0.97)	83	<0.01
Prebiotics	3	−1.50 (−3.15, 0.15)	84	<0.01
**Animal model**				
Wistar	4	−1.72 (−2.31, −1.12)	19	0.3
C57BL/6	2	−0.97 (−1.85, −0.09)	47.7	0.17
Kunming	2	−2.59 (−3.91, −1.28)	60.8	0.11
Sprague–Dawley	7	−0.76 (−1.27, −0.26)	61.8	<0.01
Other	4	−4.22 (−6.77, −1.67)	93	<0.01
**Modeling methods**				
I/R	2	−1.35 (−2.02, −0.67)	33.4	0.22
LPS	3	−2.5 (−3.64, −1.37)	52.6	0.12
D-galactosamine	5	−1.31 (−2.33, −0.28)	72.6	<0.01
t-BHP	2	−3 (−5.16, −0.84)	83	<0.01
Other	6	−1.76 (−2.80, −0.71)	84	<0.01
CCl4	4	−3.25 (−5.72, −0.79)	94.8	<0.01
**Bacterial strains**				
*Lactobacillus*	14	−1.42 (−1.98, −0.85)	79	<0.01
*Bifidobacterium*	5	−1.1 (−1.88, −0.32)	64	0.02
Other strains	8	−0.85 (−1.68, −0.01)	84	<0.01

*Subgroup analysis was conducted using random-effects model. ICR, Imprinting Control Region; I/R, Ischemia–reperfusion; TAA, Thioactamide*.

**Table 3:** Univariate meta-regression of AST with four major variables.

**_ES**	**Coef**.	**Std. Err**.	***t***	***p***	**[95% CI]**	
**(A) Type of microbial agents: probiotics/prebiotics**						
typnum	0.3258473	1.309044	0.25	0.805	−2.337422	2.989117
_cons	−2.253736	1.476211	−1.53	0.136	−5.25711	0.7496375
**(B) Beneficial bacteria used in the intervention group**						
bacnum	0.0660074	0.1736054	0.38	0.706	−0.2885421	0.420557
_cons	−2.127568	0.6572909	−3.24	0.003	−3.469935	−0.7852006
**(C) The type of animal model used in the experiment**						
aninum	−0.4717258	0.1692421	−2.79	0.009	−0.8160514	−0.1274002
_cons	−0.5419476	0.5287788	−1.02	0.313	−1.617756	0.5338609
**(D) Different modeling methods of ALI**						
modnum	−0.1017024	0.1020911	−1	0.326	−0.3094084	0.1060035
_cons	−1.365523	0.6279222	−2.17	0.037	−2.64304	−0.0880055

*The variable was considered to be the source of heterogeneity when p < 0.05*.

### Effect of Microbial Agents on Bacteria-Originated Endotoxin

**Figure 4:**
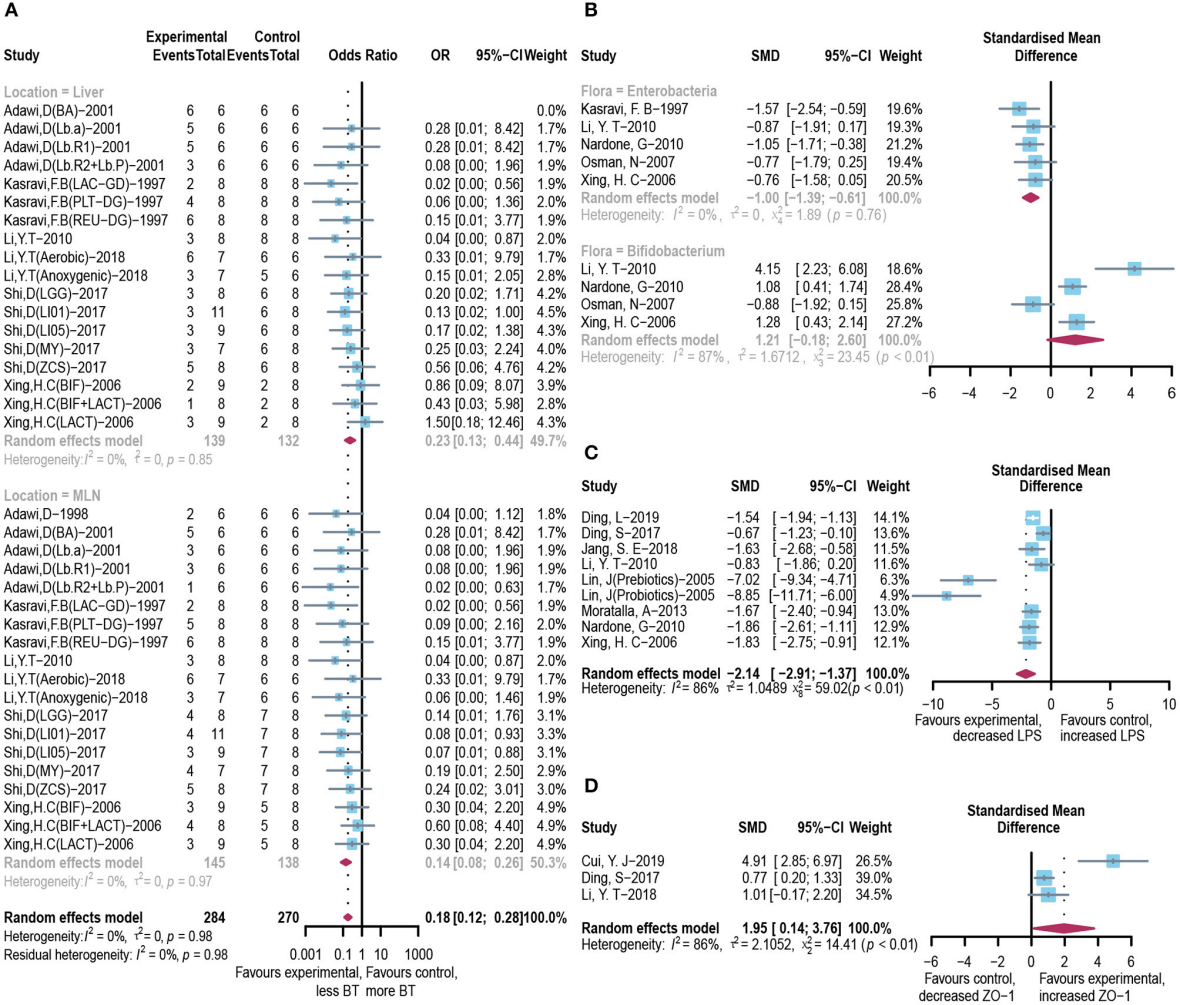
Effectiveness of microbial agents on BT. **(A)** The effect of microbial agents on BT to liver or mesenteric lymph node. **(B)** The effect of microbial agents on the abundance of *Enterococcus* and *Bifidobacterium* in the ileocecal region. **(C)** The effect of microbial agents on the level of LPS. **(D)** The effect of microbial agents on the expression of tight junction protein ZO-1.

Integrated investigation of the terminal ileum flora, BT, and endotoxin was conducted to give a detailed evaluation of the changes in gut microbiota and toxin metabolism in ALI. The incidence of BT to the liver (OR = 0.23, 95% CI: 0.13–0.44) and mesenteric lymph node (OR = 0.14, 95% CI: 0.08–0.26; Figure 4A) was significantly reduced in the microbial intervention group without heterogeneity (*I*^2^ = 0%, *p* = 0.85 and *I*^2^ = 0%, *p* = 0.97, respectively). Notably, although the horizontal lines of most of the selected studies intersect with the invalid line, which may be caused by the small sample size, the combined results showed significance. After the use of prebiotics or probiotics, the abundance of detrimental *Enterococcus* in the ileocecal region was less than that of the control group (SMD: −1.00, 95% CI: −1.39 to −0.61) with good homogeneity (*I*^2^ = 0%, *p* = 0.76), while most studies believed that the colonization of *Bifidobacterium*, a beneficial bacterium, increased in this area (SMD: 1.21, 95% CI: −0.18 to 2.60, *I*^2^ = 87%, and *p* < 0.01; Figure 4B). Accordingly, endotoxin exhibited reduced levels in the microbial treatment group (SMD: −2.14, 95% CI: −2.91 to −1.37, *I*^2^ = 86%, and *p* < 0.01; Figure 4C), with increased expression of intestinal tight junction protein ZO-1 (SMD: 1.95, 95% CI: 0.14–3.76, *I*^2^ = 86%, and *p* < 0.01) (Figure 4D), indicating an augmented gut barrier and stable intestinal permeability.

### Effect of Microbial Agents on Inflammation Mediators

**Figure 5:**
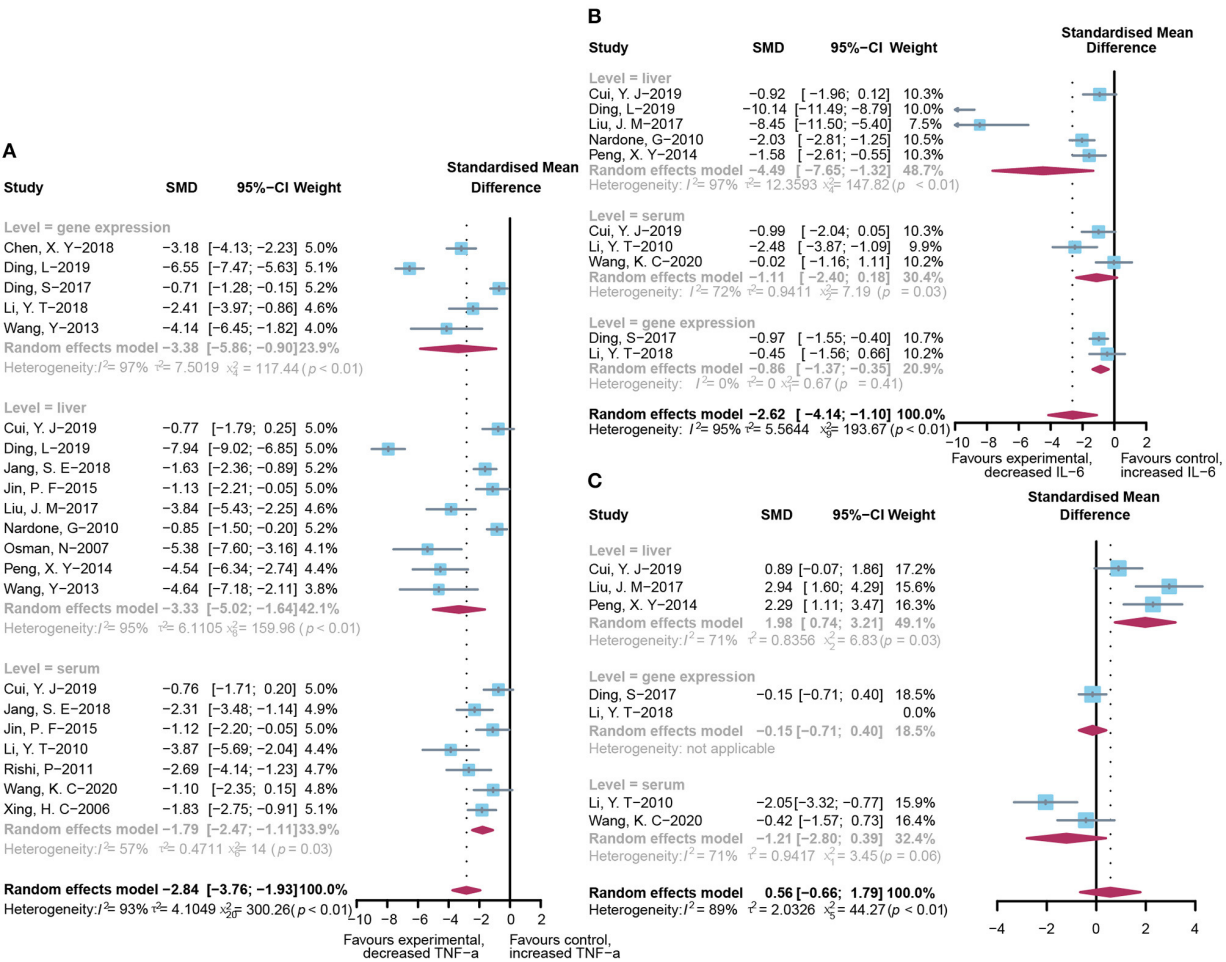
Effectiveness of microbial agents on inflammatory cytokines. **(A)** The effect of microbial agents on TNF-α. **(B)** The effect of microbial agents on IL-6. **(C)** The effect of microbial agents on IL-10.

Pooled analysis of inflammation mediators, such as TNF-α, IL-6, and IL-10, has also been applied to assess the inflammatory infiltration of the liver after the occurrence of acute injury. As shown in Figure 5, the administration of probiotics or prebiotics, compared to placebo, decreased TNF-α (SMD: −2.84, 95% CI: −3.76 to −1.93) (Figure 5A) and IL-6 (SMD: −2.62, 95% CI: −4.14 to −1.10) (Figure 5B) with significant heterogeneity (*I*^2^ = 93%, *p* < 0.01 and *I*^2^ = 95%, *p* < 0.01, respectively). However, except for significant heterogeneity (*I*^2^ = 89%, *p* < 0.01), an interesting trend was noted for the pooled result of IL-10, which showed higher levels in the microbial intervention group than in the placebo-treated group (SMD: 0.56, 95% CI: −0.66 to 1.79; Figure 5C), with the statistical combination intersected in the invalid line. To explore the sources of heterogeneity and the impact of different measurement levels on the results, inflammatory cytokines were separated into three subgroups, designated as “liver,” “serum,” and “gene expression.” Remarkably, compared to the control group, IL-10 levels increased in the liver (SMD: 1.98, 95% CI: 0.74–3.21, *I*^2^ = 71%, and *p* = 0.03) and decreased in the serum (SMD: −1.21, 95% CI: −2.80 to 0.39, *I*^2^ = 71%, and *p* = 0.06), showing the opposite trend. Subgroup analysis and meta-regression of TNF-α suggested that the animal model and modeling methods were the sources of heterogeneity (Supplementary Tables 2B, 3B). Egger's test suggested the existence of publication bias (*p* < 0.01). The contour-enhanced funnel plot with the trim-and-fill method (Figure 3C) indicates that publication bias was not the main cause of asymmetry. Sensitivity analysis confirmed the robustness of the study (Supplementary Figure 1C).

### Effect of Microbial Agents on Oxidative Stress

**Figure 6:**
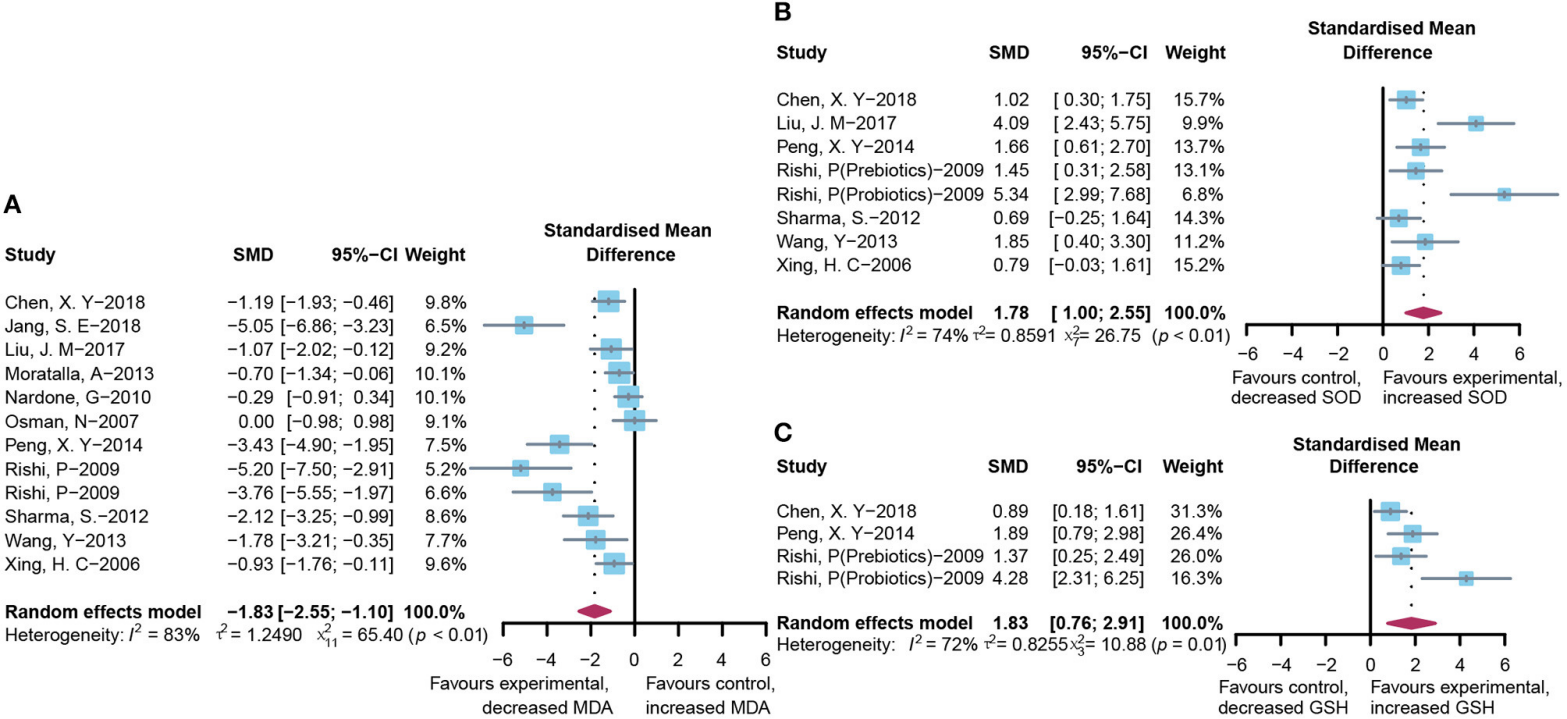
Effectiveness of microbial agents on oxidative stress. **(A)** The effect of microbial agents on MDA to evaluate free radical mediated lipid peroxidation injury. **(B,C)** The effect of microbial agents on SOD and GSH to evaluate antioxidant status of tissues.

To assess the free radical-mediated lipid peroxidation injury and antioxidant status of tissues, we combined the levels of MDA, SOD, and GSH respectively. The treatment of microbial agents contributed to the decrease in MDA level (SMD: −1.83, 95% CI: −2.55 to −1.10, *I*^2^ = 83%, and *p* < 0.01), along with the enhancement of antioxidant capacity, which was reflected in the increased level of SOD (SMD: 1.78, 95% CI: 1.00–2.55, *I*^2^ = 74%, and *p* < 0.01) and GSH (SMD: 1.83, 95% CI: 0.76–2.91, *I*^2^ = 72%, and *p* = 0.01), with obvious heterogeneity (Figures 6A–C). Subgroup analysis suggested that heterogeneity was alleviated to a certain extent after grouping according to animal model and modeling methods, suggesting that these two might be the source of heterogeneity (Supplementary Table 2C), which was further verified using meta-regression to show that the *p*-values of both were <0.05 (*p* = 0.02 and 0.03, respectively; Supplementary Table 3C). The contour-enhanced funnel plot with the trim-and-fill method (Figure 3D) indicates that publication bias was not the main cause of asymmetry. Sensitivity analysis proved the robustness of the results (Supplementary Figure 1D).

## Discussion

The crosstalk between the gut microbiota and the liver is increasingly emphasized, strengthened by the growing evidence that the alterations in composition or diversity of intestinal flora are involved in the progression and prognosis of many chronic liver diseases, including nonalcoholic fatty liver disease, liver cirrhosis, and hepatocellular carcinoma, and improvement in the gut microbiota has been shown to play a therapeutic role. The present study was designed to determine the effects of regulation of the microbiota using probiotics or prebiotics on ALI through the quantitative analysis of 26 reliable studies. To our knowledge, this is the first meta-analysis of microbial therapy for ALI.

The intimate bidirectional relationship between the gut and the liver constitutes the gut–liver axis, which also involves the biliary tract, the portal vein, the systemic circulation, and a series of systemic mediators. The gut-derived products permeate the intestinal barrier and translocate to the liver through the portal vein, thereby influencing liver function (35). Accordingly, the increased production of inflammatory cytokines during liver injury contributes to elevated systemic levels, reaching the intestine through the blood circulation and impairing intestinal mucosal immunity (36); this would, in turn, disturb the intestinal flora balance and disrupt the integrity of the gut barrier. Once this dynamic balance is disturbed under the invasion of various pathogenic factors, the dysfunction of the gut and liver is triggered. Fortunately, microbial agents, such as probiotics and prebiotics, can rectify the composition and metabolic activity of intestinal microflora, thereby restoring the homeostasis of the gut–liver axis.

The most predominant genera used in probiotic products are *Lactobacillus* and *Bifidobacterium* spp because of their antibacterial efficiency. These two beneficial bacteria compete with pathogenic bacteria for the same binding sites on intestinal epithelial cells and effectively abrogate the proliferation of pathogens by releasing antibacterial substances (37). In addition, the introduction of *Lactobacillus* and *Bifidobacterium* also contributes to the proliferation of other probiotics, resulting in the improvement in the microbial structure and abundance in specific intestinal sites. Our results showed that after the microecological intervention, with the increase in beneficial strains, such as *Bifidobacterium*, in the ileocecal area, the abundance of the harmful bacteria *Enterococcus* decreased, and correspondently, the rate of BT reduced. Besides the above classical health-promoting bacterial strains, other potential beneficial species, including *Clostridium butyricum, Bacillus*, and *Pediococcus*, which use oligosaccharides, were also promoted by prebiotics. The fermentation products of prebiotics can increase the production of mucin and regulate the action of hepatic lipogenic enzymes. As shown in our results, we observed a decline in aminotransferase and enhancement in the gut barrier compared to the control group. The prebiotics can thereby exert synergistic effects together with the stimulated beneficial bacteria in the treatment of liver injury (38).

The gut barrier plays an integral role in maintaining the homeostasis of the gut–liver axis. The progression of ALI is closely linked to deteriorated intestinal permeability, which can be restored by the administration of microbial agents. In our study, the intake of prebiotics or probiotics significantly increased the expression of the tight junction protein ZO-1 in the experimental group, indicating enhanced integrity of the gut barrier. Beneficial bacteria have been proven to produce metabolites, including secreted proteins, indole, short-chain fatty acids, and bacteriocins that enhance the gut barrier by directly promoting mucus secretion by goblet cells, increasing the release of antimicrobial peptides, or facilitating the expression of tight junction proteins. For instance, the soluble proteins p75 and p40 produced by *Lactobacillus rhamnosus* GG (LGG) transactivate the epidermal growth factor receptor (39), followed by the upregulation of a proliferation-inducing ligand in the epithelium (40), which, in turn, stimulates the secretion of immunoglobulin A and relieves cytokine-induced apoptosis in the intestinal epithelial cells (40). In addition, stimulated by p75 and p40, the intestine epithelial cells can produce heat stress proteins Hsp72 and Hsp25, which increase tight junction proteins and activate the Akt pathway in a phosphatidylinositol 3-kinase-dependent manner to enhance the survival of gut epithelial cells, thus forging an impregnable intestinal barrier to prevent BT and the invasion by toxins (41). Accordingly, we found less BT to the liver and mesenteric lymph node with a deceased level of endotoxin compared to the control group.

A serious liver injury could induce excessive partial or systemic inflammation, which may culminate in liver failure and even multiple organ dysfunction. BT and invasion of noxious entities through the portal system due to compromised gut barrier leads to the activation of Kupffer cells, followed by the combination of TLRs and LPS, resulting in the activation of mitogen-activated protein kinase (MAPK) and NF-κB that initiates the production of inflammatory cytokine TNF-α, IL-6, and IL-1β (42). In the inflammatory cascade, unsaturated fatty acids are driven by reactive oxygen species (ROS) to produce lipid peroxidase that triggers fatty acid side-chain reactions and generates MDA, which causes harm to protein and DNA, thereby disrupting basic physiological functions (43). Administration of microbial agents does not only ameliorate oxidative stress by suppressing ROS formation (44) but also significantly reduces the level of cytokines through inhibition of TLR (TLR4 and TLR5)-mediated endotoxin activation and decreases the phosphorylation of MAPK p38, thus attenuating the inflammatory response (44). Consistently, our findings suggested that probiotics or prebiotics inhibited the release of the cytokines TNF-α and IL-6, decreased the level of MDA, but increased the concentrations of the antioxidants SOD and GSH. According to subgroup analysis, the anti-inflammatory cytokine IL-10 showed an opposite trend in the liver and serum. It was reported that IL-10 plays a protective immunomodulatory role in inflammation-related liver diseases by downregulating the expression of major histocompatibility complex (MHC) II and maintaining the suppressive function of regulatory T cells; thus, the level of IL-10 in the liver is negatively correlated with the degree of liver injury (45). Since the modeling methods in most of the studies were applied locally instead of entering into the blood circulation directly, the concentration of cytokines in the liver would be high, making the increase in IL-10 in the serum relatively inconspicuous and inaccurate due to the limitation of observation time. The serum IL-10 level and the degree of liver injury appear to be positively correlated, which did not show significance in our results. Therefore, more evidence is needed to support and confirm the relationship between serum IL-10 and ALI.

The study presents several limitations. First, unavoidable heterogeneity emerged when we combined certain indicators due to the exploratory nature of animal studies, even if the random-effects model and subgroup analysis were performed. However, meta-regression indicated the source of heterogeneity, and sensitivity analysis confirmed that our results were robust. In addition, asymmetry appeared in the funnel plot, and Egger's test indicated the existence of publication bias. However, the contour-enhanced funnel plot with trim-and-fill methods ultimately demonstrated that heterogeneity was the main cause of the asymmetry, and the number of unpublished nonsignificant literature was estimated to be very small. In this context, our conclusions based on 26 articles are still of reference significance. Furthermore, we did not include symbiotics and postbiotics because the quality of the literature did not meet our expectations. Finally, we did not explore a dose–response relationship between microecological therapy and ALI efficacy because of the limited data provided by the literature.

## Conclusion

Our study demonstrated that probiotics and prebiotics have a significant ameliorative effect on ALI through the gut–liver axis by upregulating tight junction protein ZO-1, correcting the abundance of the ileum flora, reducing BT and endotoxin invasion, and suppressing oxidative stress and proinflammatory mediators, thus, in turn, improving liver biochemical indicators. Our findings also provide a novel insight into the microbial therapies for ALI in clinical practice and imply a promising prospect of applying the gut–liver axis in the management of liver diseases. More clinical studies are required to facilitate the transformation of these preclinical research results into practical applications.

## Data Availability Statement

The raw data supporting the conclusions of this article will be made available by the authors, without undue reservation.

## Author Contributions

CX and FW conceived the study idea, guided the work, checked the data, and revised the manuscript. SX designed the retrieval strategy, analyzed the data, drew the pictures, and drafted the manuscript. MZ and QW screened the studies, assessed the methodology quality, extracted study information, and made the flow charts. ZXu and BP analyzed and checked the data. YX and ZD designed retrieval strategy and beautified the pictures. SW beautified the pictures and made the tables. ZXue guided the work and revised the manuscript. All co-authors had full access to and approved the final version of the manuscript.

## Funding

This work was supported by grants from the Zhejiang Provincial Natural Science Foundation of China (LY20H180010) and Wenzhou Science and Technology Bureau (Y20180142).

## Conflict of Interest

The authors declare that the research was conducted in the absence of any commercial or financial relationships that could be construed as a potential conflict of interest.

## Publisher's Note

All claims expressed in this article are solely those of the authors and do not necessarily represent those of their affiliated organizations, or those of the publisher, the editors and the reviewers. Any product that may be evaluated in this article, or claim that may be made by its manufacturer, is not guaranteed or endorsed by the publisher.

## Abbreviations

ALI: Acute liver injury
ALT: Alanine transaminase
AST: Aspartate aminotransferase
BT: Bacterial translocation
CI: Confidence intervals
D-Ga1N: D-galactosamine
GSH: Glutathione
ICR: Imprinting Control Region
LPS: Lipopolysaccharides
MDA: Malondialdehyde
MD: Mean differences
NF-κB: Nuclear factor kappa B
OR: Odds ratio
ROS: Reactive oxygen species
SD: Standard deviations
SOD: Superoxide dismutase
TLRs: Toll-like receptors
ZO-1: Zonula occludens.
