# Supplementary material for: Effectiveness of Probiotics and Prebiotics Against Acute Liver Injury: A Meta-Analysis
Source: Front Med (Lausanne). 2021 Sep 21;8:739337. doi: 10.3389/fmed.2021.739337 (PMC8490661; doi:10.3389/fmed.2021.739337)
Supplement: Supplementary file 1 [file Data_Sheet_1.doc]

**Supplementary Material**

**Figure Legends**

**Supplementary Figure 1. Sensitivity analysis of AST, ALT, TNF-a and MDA.** (**A**) Sensitivity analysis of AST. (**B**) Sensitivity analysis of ALT. (**C**) Sensitivity analysis of TNF-α. (**D**) Sensitivity analysis of MDA. SMD = Standardized mean difference; CI = Confidence interval.
