# Supplementary material for: Effectiveness of Probiotics and Prebiotics Against Acute Liver Injury: A Meta-Analysis
Source: Front Med (Lausanne). 2021 Sep 21;8:739337. doi: 10.3389/fmed.2021.739337 (PMC8490661; doi:10.3389/fmed.2021.739337)
Supplement: Supplementary file 2 [file Data_Sheet_2.doc]

**Supplementary Table 1. Assessment of methodology quality according to** **SYRCLE****'s risk of bias tool.**

|  |  | Selection bias | | | Performance bias | | Detection bias | | Attrition bias | Reporting bias | Other |
| --- | --- | --- | --- | --- | --- | --- | --- | --- | --- | --- | --- |
| Study | Year | Sequence generation | Baseline characteristics | Allocation concealment | Random housing | Blinding | Random outcome assessment | Blinding | Incomplete outcome data | Selective outcome reporting | Other sources of bias |
| Adawi, D | 2001 | low risk | low risk | unclear | low risk | high risk | unclear | unclear | low risk | low risk | low risk |
| Adawi, D | 1998 | unclear | low risk | unclear | low risk | high risk | unclear | unclear | low risk | low risk | low risk |
| Moratalla, A | 2013 | unclear | low risk | unclear | low risk | high risk | low risk | unclear | unclear | low risk | unclear |
| Chen, X. Y | 2018 | low risk | low risk | unclear | low risk | high risk | low risk | unclear | low risk | low risk | unclear |
| Cui, Y. J | 2019 | low risk | low risk | unclear | low risk | high risk | low risk | unclear | low risk | low risk | unclear |
| Ding, S | 2017 | low risk | low risk | unclear | low risk | high risk | low risk | unclear | low risk | low risk | unclear |
| Ding, L | 2019 | low risk | low risk | unclear | low risk | high risk | low risk | unclear | low risk | low risk | unclear |
| Han, S. Y. | 2005 | unclear | low risk | unclear | low risk | high risk | unclear | unclear | low risk | low risk | low risk |
| Huang, H. P | 2019 | low risk | low risk | unclear | low risk | high risk | low risk | unclear | low risk | low risk | unclear |
| Jin, P. F | 2015 | low risk | low risk | unclear | low risk | high risk | low risk | unclear | low risk | low risk | unclear |
| Kasravi, F. B | 1997 | unclear | low risk | unclear | low risk | high risk | unclear | unclear | low risk | low risk | low risk |
| Li, Y. T | 2010 | low risk | unclear | unclear | low risk | high risk | unclear | unclear | low risk | low risk | low risk |
| Li, Y. T | 2018 | unclear | low risk | unclear | low risk | high risk | low risk | unclear | low risk | low risk | unclear |
| Lin, J | 2005 | low risk | low risk | unclear | low risk | high risk | unclear | unclear | low risk | low risk | low risk |
| Liu, J. M | 2017 | unclear | unclear | unclear | low risk | high risk | low risk | unclear | unclear | low risk | unclear |
| Nardone, G | 2010 | low risk | low risk | unclear | unclear | high risk | unclear | unclear | low risk | low risk | low risk |
| Nicaise, C | 2008 | unclear | unclear | unclear | low risk | high risk | unclear | unclear | low risk | low risk | low risk |
| Osman, N | 2007 | low risk | low risk | unclear | low risk | high risk | unclear | unclear | low risk | low risk | low risk |
| Park, J. H. | 2012 | unclear | low risk | unclear | low risk | high risk | unclear | unclear | low risk | low risk | low risk |
| Peng, X. Y | 2014 | low risk | low risk | unclear | low risk | high risk | low risk | unclear | low risk | low risk | unclear |
| Rishi, P | 2011 | unclear | low risk | unclear | low risk | high risk | unclear | unclear | low risk | low risk | low risk |
| Rishi, P | 2009 | unclear | low risk | unclear | low risk | high risk | unclear | unclear | low risk | low risk | low risk |
| Sharma, S. | 2012 | unclear | low risk | unclear | low risk | high risk | unclear | unclear | low risk | low risk | low risk |
| Wang, K. C | 2021 | low risk | low risk | unclear | low risk | high risk | low risk | unclear | low risk | low risk | unclear |
| Wang, Y | 2019 | unclear | low risk | unclear | low risk | high risk | unclear | unclear | unclear | low risk | unclear |
| Xing, H. C | 2006 | low risk | low risk | unclear | low risk | high risk | unclear | unclear | low risk | low risk | low risk |

**Supplementary Table 2 Subgroup analysis of ALT, TNF-α and MDA**

| **Supplementary Table 2A. Subgroup analysis of ALT** | | | | |
| --- | --- | --- | --- | --- |
|  | **No. of studies** | **SMD(95%CI)** | **I2(%)** | **p-value** |
| **Microbial agents** |  |  |  |  |
| Probiotics | 24 | -1.54(-2.02,-1.07) | 80 | <0.01 |
| Prebiotics | 4 | -0.71(-1.71,0.26) | 71 | 0.02 |
| **Animal model** |  |  |  |  |
| BALB/c mice | 2 | -2.78(-4.41,-1.15) | 55 | 0.13 |
| C57BL/6 | 5 | -0.63(-1.33,0.07) | 60 | 0.04 |
| ICR | 3 | -2.14(-5.26,1.00) | 94 | <0.01 |
| Kunming | 2 | -2.32(-1.82,-0.66) | 76 | 0.04 |
| Sprague-Dawley | 12 | -1.24(-1.82,-0.66) | 75 | <0.01 |
| Wistar | 4 | -2.24(-3.37,-1.11) | 70 | 0.02 |
| **Modling methods** |  |  |  |  |
| Other | 4 | -1.44(-2.34,-0.54) | 61.5 | 0.05 |
| CCl4 | 4 | -2.02(-3.86,-0.18) | 91.5 | <0.01 |
| D-galactosamine | 8 | -1.33(-2.08,-0.58) | 68.9 | <0.01 |
| I/R | 2 | -2.25(-4.09,-0.40) | 86.9 | <0.01 |
| LPS | 4 | -2.45(-3.78,-1.12) | 74.4 | <0.01 |
| S.typhimurium | 2 | -2.78(-4.41,-1.15) | 55.4 | 0.13 |
| TAA | 4 | -0.11(-0.56,0.35) | 0 | 0.56 |
| t-BHP | 2 | -1.63(-2.37,-0.88) | 0 | 0.45 |
| **Bacterial strains** |  |  |  |  |
| Lactobacillus | 18 | -1.94(-2.48,-1.41) | 79.80% | <0.01 |
| Bifidobacterium | 7 | -1.89(-2.69,-1.09) | 67.50% | <0.01 |
| Other strains | 6 | -1.44(-2.34,-0.54) | 83.70% | <0.01 |
| Subgroup anaysis was conducted using random-effects model. ICR=Imprinting Control Region; I/R=Ischemia-reperfusion; LPS=lipopolysaccharides; TAA=Thioactamide; | | | | |

| **Supplementary Table 2B. Subgroup analysis of TNF-α** | | | | |
| --- | --- | --- | --- | --- |
|  | **No. of studies** | **SMD(95%CI)** | **I2(%)** | **p-value** |
| **Expression level** |  |  |  |  |
| gene expression | 5 | -3.38(-5.86,-0.90) | 97 | <0.01 |
| liver | 9 | -3.33(-5.02,-1.64) | 95 | <0.01 |
| serum | 7 | -1.79(-2.47,-1.11) | 57 | 0.03 |
| **Animal Model** |  |  |  |  |
| C57BL/6 | 4 | -1.28(-1.73,-0.84) | 18 | 0.3 |
| Other | 3 | -3.55(-4.3,-2.81) | 0 | 0.39 |
| Sprague-Dawley | 7 | -3.7(-5.85,-1.56) | 97 | <0.01 |
| Wistar | 3 | -2.82(-4.79,-0.96) | 83 | <0.01 |
|  |  |  |  |  |
| **Modling methods** |  |  |  |  |
| CCl4 | 3 | -2.5(-4.56,-0.44) | 93 | <0.01 |
| Other | 3 | -4.45(-6.97,-2.1) | 96 | <0.01 |
| D-galactosamine | 4 | -3.04(-4.8,-1.27) | 78 | <0.01 |
| I/R | 2 | -1.28(-2.24,-0.33) | 66 | 0.09 |
| LPS | 4 | -1.65(-2.57-0.73) | 73 | <0.01 |
| **Bacterial strains** |  |  |  |  |
| Lactobacillus | 12 | -2.82(-3.94,-1.7) | 94 | <0.01 |
| Bifidobacterium | 5 | -2.12(-2.93,-1.3) | 54 | 0.04 |
| Other strains | 4 | -1.54(-2.25,-0.84) | 70 | <0.01 |
| Subgroup anaysis was conducted using random-effects model. LPS=lipopolysaccharides; I/R=Ischemia-reperfusion | | | | |

| **Supplementary Table 2C. Subgroup analysis of MDA** | | | | |
| --- | --- | --- | --- | --- |
|  | **No. of studies** | **SMD(95%CI)** | **I2(%)** | **p-value** |
| **Animal model** |  |  |  |  |
| BALB/c mice | 2 | -3.07(-5.98,-0.16) | 90.8 | <0.01 |
| other | 2 | -2.98(-6.88,0.91) | 93.1 | <0.01 |
| Kunming | 2 | -2.21(-4.4,-0.03) | 85.9 | <0.01 |
| Sprague-Dawley mice | 2 | -0.51(-1.42,0.41) | 51 | 0.15 |
| Wistar | 3 | -1.31(-2.62,0) | 79.2 | <0.01 |
| **Modling methods** |  |  |  |  |
| CCI4 | 3 | -0.95(-1.38,-0.52) | 0 | 0.59 |
| I/R | 2 | -0.55(-1.18,0.07) | 34 | 0.22 |
| Other methods | 5 | -2.91(-4.36,-1.47) | 85 | <0.01 |
| **Bacterial strains** |  |  |  |  |
| Lactobacillus | 7 | -1.92(-2.96,-0.88) | 83 | <0.01 |
| Bifidobacterium | 4 | -1(-1.92,-0.08) | 68 | 0.03 |
| Other strains | 4 | -1.82(-2.61,-1.03) | 39 | 0.18 |
| Subgroup anaysis was conducted using random-effects model. I/R=Ischemia-reperfusion | | | | |

**Supplementary Table 3 Univariate meta-regrassion of ALT, TNF-α and MDA with four major variables**

| **Supplementary Table 3A Univariate meta-regrassion of ALT with four major variables** | | | | | | |
| --- | --- | --- | --- | --- | --- | --- |
| **(a) Type of microbial agents: probiotics/prebiotics** | | | | | | |
| _ES | Coef. | Std.Err. | t | P>|t| | [95%Conf.Interval] | |
| typnum | 0.9180324 | 0.8631098 | 1.06 | 0.294 | -0.8250532 | 2.661118 |
| _cons | -2.610186 | 0.9854884 | -2.65 | 0.011 | -4.600421 | -0.6199522 |
| **(b) Beneficial bacteria used in the intervention group** | | | | | | |
| _ES | Coef. | Std.Err. | t | P>|t| | [95%Conf.Interval] | |
| bacnum | 0.1296056 | 0.1661159 | 0.78 | 0.44 | -0.2069772 | 0.4661885 |
| _cons | -2.010331 | 0.4954942 | -4.06 | 0 | -3.014297 | -1.006364 |
| **(c) The type of animal model used in the experiment** | | | | | | |
| _ES | Coef. | Std.Err. | t | P>|t| | [95%Conf.Interval] | |
| aninum | -0.0369213 | 0.1550941 | -0.24 | 0.813 | -0.3501402 | 0.2762976 |
| _cons | -1.509376 | 0.4875935 | -3.1 | 0.004 | -2.494091 | -0.5246609 |
| **(d) Different modeling methods of acute liver injury** | | | | | | |
| _ES | Coef. | Std.Err. | t | P>|t| | [95%Conf.Interval] | |
| modnum | 0.0378141 | 0.0741398 | 0.51 | 0.613 | -0.1119143 | 0.1875425 |
| _cons | -1.804444 | 0.4674638 | -3.86 | 0 | -2.748506 | -0.8603815 |
| The variable was considered to be the source of heterogeneity when P < 0.05. | | | | | | |

| **Supplementary Table 3B Univariate meta-regrassion of** **TNF-α with four major variables** | | | | | | |
| --- | --- | --- | --- | --- | --- | --- |
| **(a) Expression level of TNF-α** | | | | | | |
| _ES | Coef. | Std.Err. | t | P>|t| | [95%Conf.Interval] |  |
| levnum | 0.3129568 | 0.4472342 | 0.7 | 0.49 | -0.6017399 | 1.227653 |
| _cons | -3.199164 | 0.997993 | -3.21 | 0.003 | -5.240289 | -1.15804 |
| **(b) Beneficial bacteria used in the intervention group** | | | | | | |
| _ES | Coef. | Std.Err. | t | P>|t| | [95%Conf.Interval] |  |
| bacnum | 0.2279007 | 0.2389173 | 0.95 | 0.348 | -0.26074 | 0.7165414 |
| _cons | -3.095103 | 0.6748734 | -4.59 | 0 | -4.475374 | -1.714832 |
| **(c) The type of animal model used in the experiment** | | | | | | |
| _ES | Coef. | Std.Err. | t | P>|t| | [95%Conf.Interval] |  |
| aninum | -0.6509269 | 0.3090647 | -2.11 | 0.044 | -1.283035 | -0.0188187 |
| _cons | -1.205479 | 0.7123436 | -1.69 | 0.101 | -2.662385 | 0.251427 |
| **(d) Different modeling methods of acute liver injury** | | | | | | |
| _ES | Coef. | Std.Err. | t | P>|t| | [95%Conf.Interval] |  |
| modnum | -0.3899901 | 0.1839905 | -2.12 | 0.043 | -0.766293 | -0.0136872 |
| _cons | -1.190625 | 0.712274 | -1.67 | 0.105 | -2.647389 | 0.2661386 |
| The variable was considered to be the source of heterogeneity when P < 0.05. | | | | | | |

| **Supplementary Table 3C Univariate meta-regrassion of MDA with four major variables** | | | | | | |
| --- | --- | --- | --- | --- | --- | --- |
| **(a) Type of microbial agents: probiotics/prebiotics** | | | | | | |
| _ES | Coef. | Std.Err. | t | P>|t| | [95%Conf.Interval] | |
| typnum | -2.150926 | 1.668019 | -1.29 | 0.218 | -5.728471 | 1.426618 |
| _cons | 0.3284283 | 1.795821 | 0.18 | 0.858 | -3.523224 | 4.180081 |
| **(b) Beneficial bacteria used in the intervention group** | | | | | | |
| _ES | Coef. | Std.Err. | t | P>|t| | [95%Conf.Interval] | |
| bacnum | -0.1848986 | 0.3492841 | -0.53 | 0.605 | -0.9394811 | 0.5696838 |
| _cons | -1.425679 | 0.8649242 | -1.65 | 0.123 | -3.294234 | 0.4428761 |
| **(c) The type of animal model used in the experiment** | | | | | | |
| _ES | Coef. | Std.Err. | t | P>|t| | [95%Conf.Interval] | |
| aninum | -0.4582614 | 0.175266 | -2.61 | 0.02 | -0.8341695 | -0.0823533 |
| _cons | -0.4731078 | 0.6358067 | -0.74 | 0.469 | -1.836778 | 0.890562 |
| **(d) Different modeling methods of acute liver injury** | | | | | | |
| _ES | Coef. | Std.Err. | t | P>|t| | [95%Conf.Interval] | |
| modnum | -0.4599716 | 0.127648 | -3.6 | 0.003 | -0.7337494 | -0.1861938 |
| _cons | 0.111805 | 0.602093 | 0.19 | 0.855 | -1.179556 | 1.403166 |
| The variable was considered to be the source of heterogeneity when P < 0.05. | | | | | | |
