# Supplementary figures and images for: Effectiveness of Probiotics and Prebiotics Against Acute Liver Injury: A Meta-Analysis
Source: Front Med (Lausanne). 2021 Sep 21;8:739337. doi: 10.3389/fmed.2021.739337 (PMC8490661; doi:10.3389/fmed.2021.739337)

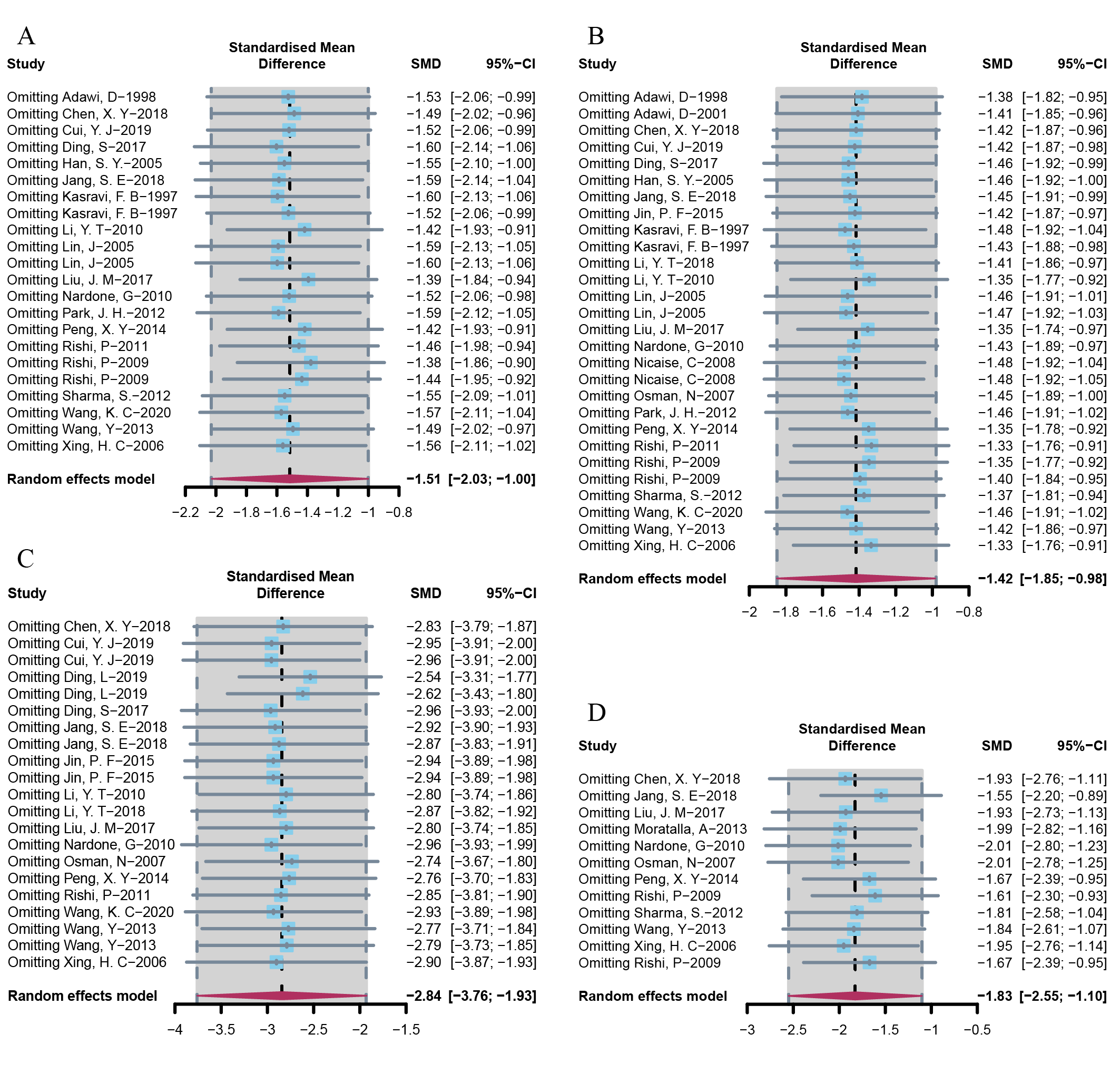

Supplement: Supplementary file 3 [file Image_1.TIF]
